# The Projection From Ventral CA1, Not Prefrontal Cortex, to Nucleus Accumbens Core Mediates Recent Memory Retrieval of Cocaine-Conditioned Place Preference

**DOI:** 10.3389/fnbeh.2020.558074

**Published:** 2020-11-16

**Authors:** Yiming Zhou, Enhui Yan, Deqin Cheng, Huiwen Zhu, Zhiyuan Liu, Xi Chen, Lan Ma, Xing Liu

**Affiliations:** The State Key Laboratory of Medical Neurobiology and MOE Frontiers Center for Brain Science, School of Basic Medical Sciences and the Institutes of Brain Science, Fudan University, Shanghai, China

**Keywords:** memory retrieval, cocaine, conditioned place preference, nucleus accumbens, hippocampus

## Abstract

Drug-paired cues inducing memory retrieval by expressing drug-seeking behaviors present a major challenge to drug abstinence. How neural circuits coordinate for drug memory retrieval remains unclear. Here, we report that exposure of the training chamber where cocaine-conditioned place preference (CPP) was performed increased neuronal activity in the core of nucleus accumbens (AcbC), ventral CA1 (vCA1), and medial prefrontal cortex (mPFC), as shown by elevated pERK and c-Fos levels. Chemogenetic inhibition of neuronal activity in the vCA1 and AcbC, but not mPFC, reduced the time spent in the cocaine-paired compartment, suggesting that the vCA1 and AcbC are required for the retrieval of cocaine-CPP memory and are key nodes recruited for cocaine memory storage. Furthermore, chemogenetic inhibition of the AcbC-projecting vCA1 neurons, but not the AcbC-projecting mPFC neurons, decreased the expression of cocaine-CPP. Optogenetic inhibition of the vCA1–AcbC projection, but not the mPFC–AcbC projection, also reduced the preference for the cocaine-paired compartment. Taken together, the cue-induced natural recall of cocaine memory depends on vCA1–AcbC circuits. The connectivity from the vCA1 to the AcbC may store the information of the cue–cocaine reward association critically required for memory retrieval. These data thus provide insights into the neural circuit basis of retrieval of drug-related memory.

## Introduction

The persistent and compulsive behavioral patterns associated with drug addiction depend largely on the ability of the drugs that produce powerful associative memories, linking their intense, euphorigenic properties with neutral cues. The associative memory is the critical biological basis of drug addiction (Boening, 2001; Hyman, 2005). The cue-elicited craving for drug use presents a major challenge to abstinence. The attempts to reduce cue-induced retrieval of drug memory are efficacious in the treatment of drug addiction (Kelley, 2004).

The nucleus accumbens (Acb) has a critical role in the learning and expression of addictive behaviors induced by drugs. Specifically, the core of Acb (AcbC) is a key node in limbic neural circuits that are critically involved in drug sensitization and relapsing behaviors (Xu et al., 2009, 2011; Spencer et al., 2014). Lesions and biochemical studies in animals have suggested that the AcbC also plays an essential role in the expression of cue–drug/food associations in instrumental conditioning (Parkinson et al., 1999; Corbit et al., 2001).

The Acb is a point of convergence for several afferents, including dopaminergic afferents arising from the ventral tegmental area (VTA) and glutamatergic afferents arising from the limbic and cortical regions, including the ventral hippocampus (vHipp), medial prefrontal cortex (mPFC), and basolateral amygdala (BLA; Phillipson and Griffiths, 1985; Russo and Nestler, 2013). It is thought that reward-seeking is facilitated by integrating the reinforcement signals mediated by dopamine release in the Acb with environmental stimuli mediated by glutamate innervation (Phillips et al., 2003; Stuber et al., 2008; Flagel et al., 2011). The glutamatergic circuits that converge on the spiny neurons of the Acb encode the context, cues, and descriptive features when presented with reward (Berke and Hyman, 2000; Everitt and Wolf, 2002; Kelley, 2004; Pennartz et al., 2011). Studies also suggest that β-adrenergic receptor (β-AR) activation in the mPFC, but not BLA, is required for the retrieval of cocaine-conditioned place preference (CPP) memory (Otis et al., 2013). Dopamine receptor and glycogen synthase kinase-3β-dependent signaling in the vHipp are required for the retrieval of morphine and cocaine-CPP memory (Wang et al., 2019; Barr et al., 2020). These studies suggest that mPFC, BLA, and vHipp have distinct roles in drug memory retrieval. Pathway-specific activation of these glutamate inputs to the Acb has been demonstrated to trigger different physiological responses (Goto and Grace, 2008; Sesack and Grace, 2010), while their roles in drug memory storage are largely unknown. In our previous study, we found that the preferential synaptic strengthening of the vCA1–AcbC engram circuit evoked by cocaine conditioning mediated the cocaine-CPP memory storage (Zhou et al., 2019). However, the essential circuits, such as vCA1–AcbC and mPFC–AcbC circuits, involved in environmental cue-induced retrieval of drug memory remain unclear.

To investigate the neural circuits underlying the memory retrieval of cocaine associative learning, we used cocaine-CPP as the behavioral model and applied pERK immunoblotting and c-Fos immunostaining to test the neuronal activities in the vCA1, AcbC, and mPFC after re-exposure to the cocaine associative environment. Then, we applied chemogenetic and optogenetic methods to study the roles of these brain regions and vCA1–AcbC and mPFC–AcbC projections in the environmental cue-induced retrieval of cocaine-CPP memory.

## Materials and Methods

### Animals

Male C57BL/6J mice at 6–10 weeks old (Shanghai Laboratory Animal Center, CAS) and Ai14 male mice [*Gt*(*ROSA*)*26Sortm14*(*CAG-tdTomato*)*Hze*, #007914, from The Jackson Laboratory] were housed under standard conditions of a reversed 12-h light–dark cycle with access to food and water *ad libitum*. Male mice at 8–10 weeks of age were used for all behavioral experiments carried out during their light cycle. All animal treatments were strictly in accordance with the National Institutes of Health’s Guide for the Care and Use of Laboratory Animals and approved by the Animal Care and Use Committee of Shanghai Medical College of Fudan University.

### AAV Preparation and Reagents

The AAVs prepared with a titer exceeding 2 × 10^12^ vector genome (vg) per milliliter were used for infection. *AAV_2_-EF1α-hM4D-mCitrine* was purchased from the University of North Carolina at Chapel Hill Vector Core. *AAV_9_-hSyn-WGA-Cre-mCherry*, *AAV_9_-EF1α-DIO-hM4D-GFP*, *AAV_9_-CaMKII-Cre*, *AAV_9_-hSyn-DIO-hM4D-mCherry*, *AAV_9_-EF1α-eNpHR3.0-eYFP*, and* scAAV_2/1_-hSyn-Cre* were generated and packaged by Taitool Biological.

Cocaine-hydrochloride (Qinghai Pharmaceutical Firm) was dissolved in saline at 2.5 mg/ml and injected intraperitoneally (10 mg/kg, i.p.) for cocaine-CPP conditioning. Clozapine-*N*-oxide (CNO, Sigma, #C0832) was dissolved in saline at 0.2 mg/ml and injected intraperitoneally (1 mg/kg, i.p.) 30 min before memory retention test 1.

### Stereotaxic Surgeries

For virus injection, the mice were anesthetized with isoflurane (3.5% induction, 1.5–2% maintenance), placed in a stereotaxic apparatus (Stoelting Instruments, Wood Dale, IL, USA), and injected with AAV into the vCA1 (150 nl), AcbC (150 nl), or mPFC (150 nl) using a 10-μl syringe and a 36-gauge blunt needle under the control of a UMP3 ultra microsyringe pump (World Precision Instruments, Sarasota, FL, USA). The needle was left for an additional 10 min after virus injection and was then slowly removed.

AAVs were bilaterally injected into the vCA1 [anterior–posterior (AP): −3.5 mm; medial–lateral (ML): ± 3.7 mm; dorsal–ventral (DV): −3.7 mm], AcbC (AP: +1.6 mm; ML: ± 1.2 mm; DV: −4.3 mm), and mPFC (AP: +1.8 mm; ML: ± 0.3 mm; DV: −2.2 mm) according to the respective coordinates relative to Bregma. For the optogenetic experiments, the ceramic optic fibers [200 μm in diameter, 0.37 numerical aperture (NA), Anilab Software and Instruments] were bilaterally implanted above the AcbC (AP: +1.6 mm; ML: ± 1.7 mm; DV: −4.0 mm, with 10° angle) and were fixed by a layer of dental cement. The mice remained on a heating pad until fully recovered from anesthesia after surgery and were allowed to recover for 2–3 weeks before all subsequent behavioral experiments.

### Cocaine-Conditioned Place Preference Test

The CPP apparatus (15 × 30 × 20 cm^3^) consisted of two equal-sized compartments with distinctly patterned walls, ceilings, and floorings, separated by a removable board during cocaine-CPP conditioning. One compartment was decorated with black-and-white striped walls, a white frosted floor, and a black ceiling. The other compartment had black-and-white checkered walls, a black smooth floor, and a white ceiling. A standard cocaine-CPP paradigm was applied, including pre-test, conditioning, and memory retention tests. One compartment was randomly designated as the cocaine compartment and the other as the saline compartment. Half of the mice were conditioned with cocaine in the left compartment, and the other half were conditioned with cocaine in the right compartment. During the pre-test session, the mice were released from the middle of the CPP apparatus and allowed free access and exploration to both compartments (15 min). The time spent in each chamber was recorded by a trained observer blinded to the experimental group with a stopwatch, according to the video. Mice that spent >65% (>585 s) or <35% (<315 s) of the total time (900 s) in one chamber were excluded from the subsequent CPP sessions. In this study, 249 mice were used, and 244 mice were subjected to cocaine-CPP paradigm. In total, 11 mice with a difference of more than 270 s in one compartment in the pretest were eliminated from the following procedures, and eight mice were excluded for the inaccurate optic fiber implantation and virus infection. During the conditioning session, the mice were immediately introduced into one compartment and allowed to explore for 30 min after injection with saline (4 ml/kg, i.p.). After a 6-h interval, the mice were injected with cocaine (10 mg/kg, i.p.) and placed in the other compartment for 30 min in the afternoon. The mice were injected with cocaine or saline on alternating morning and afternoon sessions with a 6-h interval and repeated for 3 days. The memory retention tests were performed as in previous sessions. At 1 day after cocaine-CPP training, the mice were moved to the experiment room and given an injection of saline (4 ml/kg, i.p.), and then the mice were immediately placed in the CPP apparatus to allow free access to both chambers (15 min). For the immunohistochemistry experiments, the mice of the non-retrieval group were moved to the experiment room and injected with saline similarly to the mice of the retrieval group except that they were not exposed to the CPP apparatus. The CPP score was calculated by subtracting the time spent in the saline-paired compartment from the cocaine-paired compartment.

### Chemogenetic and Optogenetic Manipulations

The memory retention test for chemogenetic stimulation was 15 min, and the test for optogenetic stimulation is 5 min. In our pilot experiment, 15-min optical stimulation of the vCA1–AcbC projection significantly decreased the CPP score in both test 1 and test 2. It seems that the 15-min optical inhibition might strengthen the extinction or induce an irreversible impairment of CPP memory. Then, we reduced the length of CPP test to 5 min for optogenetic stimulation. For chemogenetic inhibition, CNO (1 mg/kg, i.p.) or saline (5 ml/kg, i.p.) was injected 30 min before memory retention test 1. For optical inhibition, a yellow laser (594 nm, 5 mW, 5 min) was turned on immediately before placing the mice into the chamber and delivered during memory retention test 1. At 24 h later, the mice were re-introduced into the CPP apparatus and allowed to explore both chambers freely for 15 min with no interruption (designated as memory retention test 2).

### Brain Lysate Preparation and Western Blotting

The mice were sacrificed 15 min after the CPP memory retention test, and the brains were removed immediately. The vCA1, AcbC, and mPFC were dissected within 5 min on ice with a mouse matrix (RWD, #68707). Tissue samples were homogenized with radioimmunoprecipitation assay lysis buffer (Beyotime, #P0013B), incubated on ice (30 min), and then centrifuged at 1,000× *g* for 30 min at 4°C. The supernatants of the samples were assayed with BCA Protein Assay Kit (Thermo Fisher Scientific, #23227) and then diluted to an equal protein concentration of 2.0 μg/μl. The samples were further diluted 1:5 in SDS–PAGE loading buffer (Beyotime, #P0015F) and heated for 5 min at 95°C. Thirty micrograms of protein per aliquot was loaded onto 10% SDS polyacrylamide gels. The proteins were then transferred onto nitrocellulose membranes. The membranes were blocked with 5% nonfat milk in Tris-buffered saline (TBS) for 1 h at room temperature, probed with primary antibody [1:1,000, anti-pERK (Cell Signaling Technology, #9101S); 1:2,000, anti-ERK (Cell Signaling Technology, #4696S); and 1:2,000, anti-GAPDH (Santa Cruz Biotechnology, #sc-365062)] at 4°C overnight and then incubated with IRDye 700DX or 800DX-conjugated anti-rabbit or anti-mouse IgG (1:50,000, Rockland Immunochemicals Inc.) for 1 h at room temperature. The membranes were rinsed in TBS with Tween 20 (0.1%) and scanned in the appropriate channels (Odyssey, LI-COR Biosciences). The immunoblots were analyzed with ImageJ to measure the optical density of the bands of pERK, ERK, and GAPDH. ERK activation (relative pERK/ERK levels) was calculated by normalizing the intensity of pERK to the total ERK expression. Total ERK levels were calculated by normalizing the intensity of ERK to GAPDH expression. The data of pERK/ERK and ERK levels for each group were presented as the ratio of the averaged values in the control group.

### Immunofluorescence

All animals were sacrificed 60 min after memory retrieval for c-Fos immunostaining. The mice were anesthetized with isoflurane (3.5%) and transcardially perfused with 0.9% saline (10 min), followed by 4% paraformaldehyde (PFA; dissolved in 0.1 M Na_2_HPO_4_/NaH_2_PO_4_ buffer, pH 7.5; 5 min). The brains were removed immediately after perfusion, post-fixed in 4% PFA at 4°C (4 h), and then cryoprotected with 30% sucrose/phosphate-buffered saline (PBS) solution for several days. The brains were sectioned into 30-μm-thick slices by a vibratome (Leica) and then stored in cryoprotective buffer at −20°C. For immunofluorescent staining, each slice was washed three times in PBS, followed by incubation with a primary antibody at 4°C overnight. After rinsing in PBS, the brain slices were incubated with fluorescence-conjugated secondary antibody for 2 h at room temperature. Finally, the slices were coverslipped on the anti-quenching mounting medium (Thermo Fisher Scientific). For the primary antibody, we applied the antibody against c-Fos (rabbit, 1:2,000; Santa Cruz, #sc-52). For the secondary antibody, we used Alexa-488 IgG (mouse, 1:50,000; Jackson ImmunoResearch).

### c-Fos-Positive Cell Counting

The c-Fos-positive cells in each brain region were counted from five coronal slices per mouse. Automated cell counting was conducted by Image-Pro Plus 6.0. The boundaries of each brain region were outlined as a region of interest (ROI) according to the mouse brain atlas, and the area was quantified by applying scale calibration. The number of c-Fos-positive cells per section was calculated by applying the same threshold above background fluorescence. We divided the number of positive cells by the area of ROI as c-Fos^+^ cell counts per square millimeter. All counting experiments were conducted by a trained observer blinded to the experimental group.

### Statistical Analysis

The experimental data were presented as mean ± SEM, analyzed by SPSS, and plotted by Graphpad. Comparisons of distribution data between two groups were analyzed using Kolmogorov–Smirnov tests. The behavioral results of cocaine-CPP were analyzed with two-way repeated-measures (RM) ANOVA, followed by Bonferroni’s *post hoc* tests, with sessions (pretest, test 1, and test 2) as a within-subject factor and drug treatment or optical stimulation as a between-subjects factor. Quantification of immunostaining or Western blotting was analyzed with two-tailed Student’s *t*-test or Mann–Whitney rank sum test if the data were non-normally distributed. *P* < 0.05 was considered statistically significant.

## Results

### Cocaine-CPP Memory Retrieval Induces Neuronal Activation in the vCA1, AcbC, and mPFC

**Figure 1:**
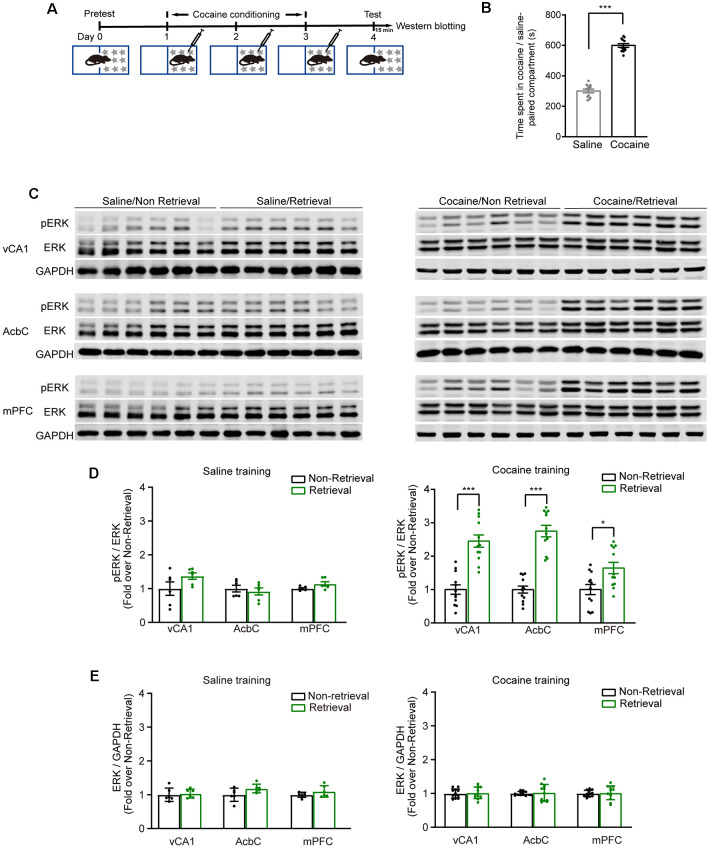
The expression levels of pERK increased in the vCA1, AcbC, and medial prefrontal cortex (mPFC) after retrieval of cocaine-conditioned place preference (CPP) memory. **(A)** Experiment scheme. At 24 h after cocaine conditioning, memory retention test was performed. The mice of the retrieval group were subjected to the memory retention test and re-exposed to the CPP apparatus. The mice of the non-retrieval group remained in their home cage. Brain tissue was collected 15 min after memory retrieval for Western blotting. **(B)** Bar graph representing the time spent in the cocaine-paired and the saline-paired compartments of the retrieval group in the memory retention test. ***P < 0.001 vs. saline-paired compartment. **(C)** Representative images of pERK expression. **(D)** Quantification of pERK levels in brain regions as indicated. **(E)** Quantification of ERK levels in brain regions as indicated. *P < 0.05, ***P < 0.001 vs. the non-retrieval group (saline conditioning groups:* n* = 6; cocaine conditioning groups: *n* = 12).

**Figure 2:**
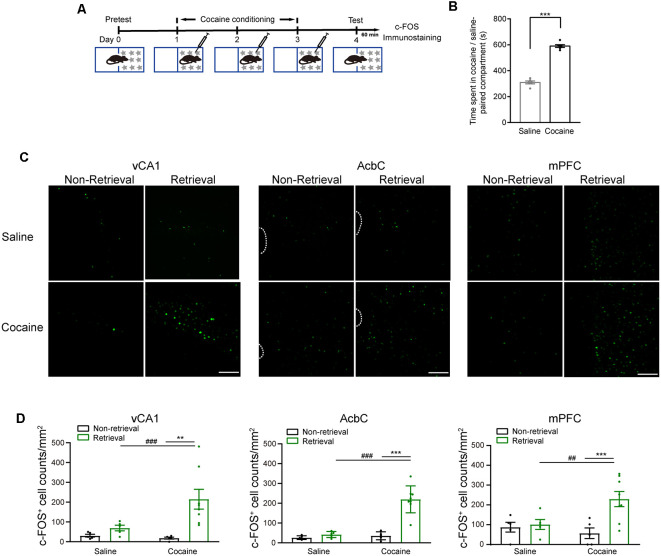
c-Fos fluorescence increased in the vCA1, AcbC, and mPFC after retrieval of cocaine-CPP memory. **(A)** Experiment scheme. At 24 h after cocaine conditioning, memory retention test was performed. The mice of the retrieval group were subjected to the memory retention test and re-exposed to the CPP apparatus. The mice of the non-retrieval group remained in their home cage. The mice were perfused 60 min after memory retrieval for c-Fos immunostaining. **(B)** Bar graph representing the time spent in the cocaine-paired and the saline-paired compartments of the retrieval group in the memory retention test. ***P < 0.001 vs. saline-paired compartment. **(C)** Representative images showing c-Fos fluorescence. Scale bar: 100 μm. **(D)** Quantification of c-Fos^+^ cell counts in brain regions as indicated. **P < 0.01, ***P < 0.001 vs. the non-retrieval group; ^##^*P* < 0.01, ^###^*P* < 0.001 vs. the saline group (saline/non-retrieval, *n* = 5; saline/retrieval, *n* = 5; cocaine/non-retrieval *n* = 5; cocaine/retrieval *n* = 8).

Extracellular signal-regulated kinase (ERK) 1 and 2, more specifically ERK 2, were activated by most drugs of abuse (Berhow et al., 1996; Pierce et al., 1999; Valjent et al., 2000; Mizoguchi et al., 2004). The mice were subjected to 3-day cocaine conditioning. At 1 day later, memory retention tests were performed (Figure 1A). The mice showed a significantly increased duration in the cocaine-paired compartment (Figure 1B, *t*_(11)_ = −12.065, *P* < 0.001, two-tailed paired Student’s *t*-test). The phosphorylation levels of ERK (pERK) were examined in the vCA1, AcbC, and mPFC 15 min after the memory retention test as memory retrieval (Figure 1C). The Western blotting data showed that the pERK levels in the vCA1, AcbC, and mPFC were significantly enhanced 15 min after memory retrieval of cocaine conditioning (Figure 1D, saline training: vCA1, *t*_(10)_ = −1.695, *P* = 0.121; AcbC, *t*_(10)_ = 0.572, *P* = 0.580; mPFC, *t*_(10)_ = −2.147, *P* = 0.057, two-tailed Student’s *t*-test; cocaine training: vCA1, *t*_(22)_ = −6.288, *P* < 0.001, two-tailed Student’s *t*-test; AcbC, *Z* = −4.157, *P* < 0.001; mPFC, *Z* = −2.194, *P* = 0.028, Mann–Whitney rank sum test). Total ERK levels were unchanged after memory retrieval (Figure 1E, saline training: vCA1, *t*_(10)_ = −0.364, *P* = 0.724; AcbC, *t*_(10)_ = −1.943, *P* = 0.081; mPFC, *t*_(10)_ = −1.318, *P* = 0.217, two-tailed Student’s *t*-test; cocaine training: vCA1, *t*_(22)_ = −0.293, *P* = 0.772, two-tailed Student’s *t*-test; AcbC, *Z* = 143, *P* = 0.707; mPFC, *Z* = 137, *P* = 0.470, Mann–Whitney rank sum test). It is reported that pERK translocates to the nucleus where it can induce the transcription of the coding sequence of *c-fos* (Chen et al., 1992; Cohen and Greenberg, 2008). c-Fos, the protein of the protooncogene *c-fos*, has been used as a marker for strongly activated neurons after drug exposure or a learning process (Crombag et al., 2002; Cruz et al., 2015). To verify the results above, c-Fos levels were measured 1 h after the memory retention test (Figure 2A). Cocaine conditioning significantly increased the preference for the cocaine-paired compartment (Figure 2B, t_5_ = 13.012, *P* < 0.001, two-tailed paired Student’s *t*-test). The c-Fos-positive cell counts in the vCA1, AcbC, and mPFC in the retrieval group of cocaine conditioning were significantly greater than those in the non-retrieval group (Figures 2C,D; vCA1: *F*_training × retrieval (1,19)_ = 4.499, *P* = 0.047; AcbC: *F*_training × retrieval (1,19)_ = 20.846, *P* < 0.001; mPFC: *F*_training × retrieval (1,19)_ = 5.326, *P* = 0.032, two-way ANOVA). These data showed that the retrieval of cocaine-CPP memory increased the neuronal activities in the vCA1, AcbC, and mPFC.

### The AcbC and vCA1 Are Required for the Retrieval of Cocaine-CPP Memory

**Figure 3:**
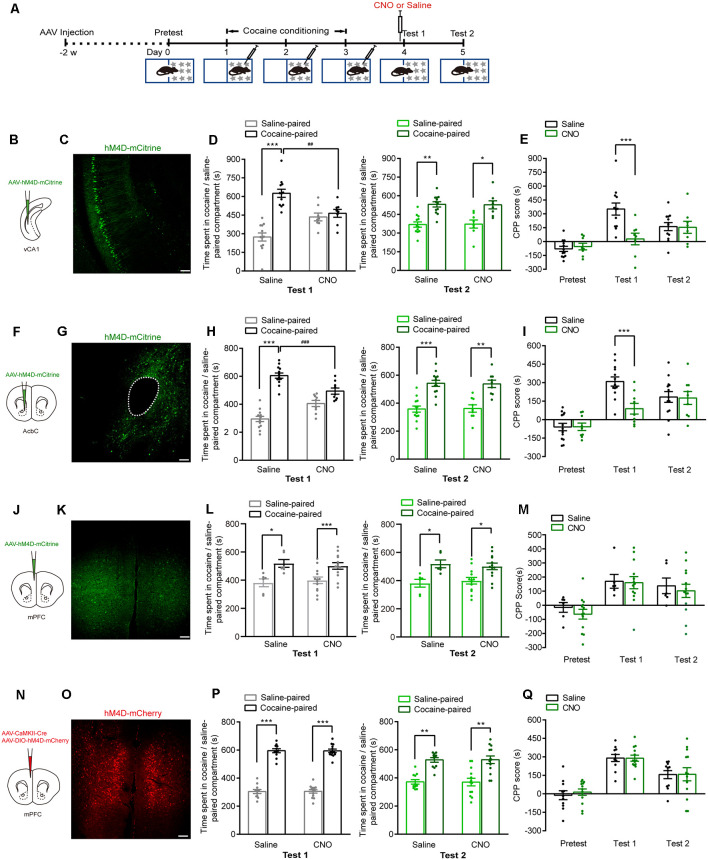
Chemogenetic inhibition of either vCA1 or AcbC neurons suppressed the retrieval of cocaine-CPP memory. **(A)** Behavioral schedule. *AAV-EF1α-hM4D-mCitrine* was injected in the vCA1, AcbC, or mPFC or *AAV-CaMKII-Cre* and *AAV-hSyn-DIO-hM4D-mCherry* were injected in the mPFC 2 weeks before CPP training. Saline or Clozapine-*N*-oxide (CNO; 1 mg/kg, i.p.) was injected 30 min before test 1. **(B,F,J,N)** Viral injection. **(C,G,K,O)** Representative images of hM4D-mCitrine or hM4D-mCherry fluorescence in vCA1 **(C)**, AcbC **(G)**, and mPFC **(K,O)**. Scale bar: 100 μm. **(D,H,L,P)** Bar graphs representing the time spent in the cocaine-paired and the saline-paired compartments of saline- and CNO-treated groups in the test 1 and test 2 sessions. *P < 0.05, **P < 0.01, ***P < 0.001 vs. the saline-paired compartment, ^##^*P* < 0.01, ^###^*P* < 0.001 vs. the saline group. **(E,I,M,Q)** Bar graphs showing the CPP scores of saline- and CNO-treated groups. ***P < 0.001 vs. saline group (vCA1: saline *n* = 12, CNO *n* = 8; AcbC: saline *n* = 13, CNO *n* = 9; mPFC: saline *n* = 6, CNO *n* = 13; mPFC: saline *n* = 11, CNO *n* = 13).

**Figure 4:**
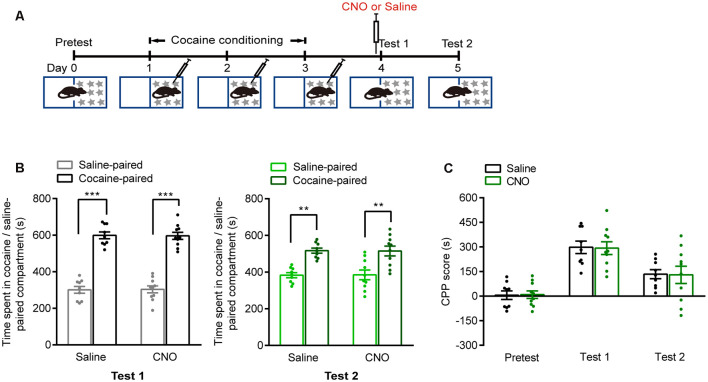
CNO treatment did not suppress the retrieval of cocaine-CPP memory. **(A)** Behavioral schedule. Saline or CNO (1 mg/kg, i.p.) was injected 30 min before test 1. **(B)** Bar graphs representing the time spent in the cocaine-paired and the saline-paired compartments of saline- and CNO-treated groups in test 1 and test 2 sessions. **P < 0.01, ***P < 0.001 vs. saline-paired compartment. **(C)** Bar graph showing the CPP scores of saline- and CNO-treated groups (Saline *n* = 9, CNO *n* = 10).

We next investigated whether these brain regions were involved in the retrieval of cocaine-CPP memory. AAVs were injected 2 weeks before cocaine conditioning. The expression of hM4D-mCitrine in the vCA1, AcbC, or mPFC or the expression of hM4D-mCherry in the mPFC glutamatergic neurons was detected (Figures 3A–C,F,G,J,K,N,O). Cocaine conditioning significantly increased the preference for the cocaine-paired compartment (Figures 3D,H,L,P). Clozapine-*N*-oxide (CNO, 1 mg/kg, i.p.) injected 30 min before test 1 significantly decreased the time spent in the cocaine-paired compartment in memory retention test 1 in vCA1:hM4D and AcbC:hM4D mice, but not in mPFC:hM4D mice (Figure 3D, test 1: *F*_treatment × compartment (1,18)_ = 11.497, *P* = 0.003; Figure 3E, *F*_treatment × session (2,36)_ = 6.637, *P* = 0.004; Figure 3H, test 1: *F*_treatment × compartment (1,20)_ = 14.834, *P* < 0.001; Figure 3I, *F*_treatment × session (2,40)_ = 7.182, *P* = 0.002; Figure 3L, test 1: *F*_treatment × compartment (1,17)_ = 0.017, *P* = 0.897; Figure 3M, mPFC:* F*_treatment × session (2,34)_ = 0.11, *P* = 0.896; Figure 3P, test 1: *F*_treatment × compartment (1,22)_ = 0.003, *P* = 0.958; Figure 3Q, *F*_treatment × session (2,44)_ = 0.101, *P* = 0.904, two-way RM ANOVA). These results showed that chemogenetic inhibition of AcbC or vCA1 neurons, but not mPFC neurons, decreased the preference for cocaine-paired chamber in memory retention test 1, indicating that inhibition of vCA1 or AcbC neuron activity impairs the retrieval of cocaine-CPP memory. The mice were re-exposed to the CPP apparatus 24 h later (test 2, without CNO treatment). The vCA1:hM4D, AcbC:hM4D, and mPFC:hM4D mice showed similar preference scores for the cocaine-paired compartment in test 2, suggesting that the chemogenetic inhibition was transient and the stability of memory storage was not affected by this interruption (Figure 3D, test 2: *F*_treatment × compartment (1,18)_ = 0.009, *P* = 0.924; Figure 3H, test 2: *F*_treatment × compartment (1,20)_ = 0.015, *P* = 0.905; Figure 3L, test 2: *F*_treatment × compartment (1,17)_ = 0.201, *P* = 0.659; Figure 3P, test 2: *F*_treatment × compartment (1,22)_ = 0.002, *P* = 0.965, two-way RM ANOVA). CNO did not affect the retrieval of cocaine-CPP memory in C57BL/6J mice that did not receive any viral infusion (Figure 4A), suggesting that no significant off-target effects of CNO were found at the dose of 1 mg/kg (Figure 4B, test 1: *F*_treatment × compartment (1,17)_ = 0.01, *P* = 0.922; test 2: *F*_treatment × compartment (1,17)_ = 0.004, *P* = 0.949; Figure 4C, *F*_treatment × session (2,34)_ = 0.008, *P* = 0.992, two-way RM ANOVA). These results indicate that the AcbC and vCA1 are critically involved in the retrieval of cocaine-CPP memory.

### The AcbC-Projecting vCA1 Neurons Are Essential for the Retrieval of Cocaine-CPP Memory

**Figure 5:**
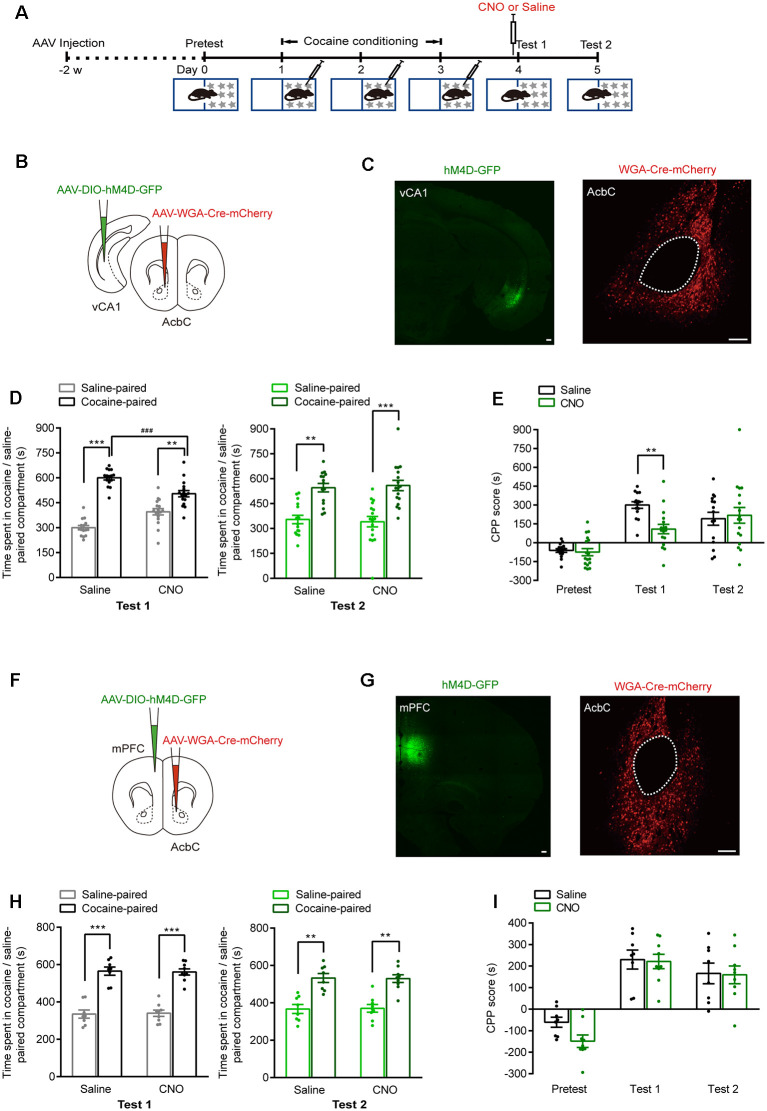
Chemogenetic inhibition of AcbC-projecting vCA1 neurons suppressed the retrieval of cocaine-CPP memory. **(A)** Behavioral schedule. *AAV-hSyn-WGA-Cre-mCherry* and *AAV-EF1α-DIO-hM4D-GFP* were injected in the indicated brain regions 2 weeks before CPP training. Saline or CNO (1 mg/kg, i.p.) was injected 30 min before test 1. **(B,F)** AAV injection strategy. **(C,G)** Representative images of hM4D-GFP and WGA-Cre-mCherry fluorescence in brain regions as indicated. Scale bar: 100 μm. **(D,H)** Bar graphs representing the time spent in the cocaine-paired and saline-paired compartment of saline- and CNO-treated groups in test 1 and test 2 sessions. **P < 0.01, ***P < 0.001 vs. saline-paired compartment, ^###^*P* < 0.001 vs. saline group. **(E,I)** Bar graphs showing the CPP scores of saline- and CNO-treated groups. **P < 0.01 vs. saline group (vCA1–AcbC: saline *n* = 15, CNO *n* = 17; mPFC–AcbC: saline *n* = 8, CNO *n* = 9).

The vCA1 and mPFC are major upstream brain regions of the AcbC. We injected anterograde self-complementary AAV (*scAAV*)-*hSyn-Cre* into the vCA1 of Ai14 mice and found that the vCA1 extensively innervated both the AcbC and the AcbSh (Supplementary Figure 1). Next, we examined the role of AcbC-projecting vCA1 neurons or mPFC neurons in the retrieval of cocaine-CPP memory (Figure 5A). *AAV-hSyn-WGA-Cre-mCherry* was injected in the AcbC, and *AAV-EF1α-DIO-hM4D-GFP* was injected in the vCA1 or mPFC. Wheat germ agglutinin (WGA) is a transsynaptic tracer that can be retrogradely taken up by synaptically connected neurons. The expression of a WGA and Cre recombinase fusion protein in the AcbC allowed Cre-dependent hM4D expression in the vCA1 or mPFC. The expression of hM4D-GFP in the AcbC-projecting vCA1 or mPFC neurons was detected (Figures 5B,C,F,G). The data showed that cocaine conditioning significantly increased the preference for the cocaine-paired compartment (Figures 5D,H). CNO (1 mg/kg, i.p.) treatment 30 min before test 1 significantly decreased the time spent in cocaine-paired compartment in memory retention test 1 in vCA1–AcbC:hM4D mice (Figure 5D, test 1: *F*_treatment × compartment (1,30)_ = 16.682, *P* < 0.001; Figure 5E, *F*_treatment × session (2,60)_ = 5.676, *P* = 0.006, two-way RM ANOVA), while CNO treatment did not change the preference for the cocaine-paired compartment or CPP scores in mPFC–AcbC:hM4D mice (Figure 5H, test 1: *F*_treatment × compartment (1,15)_ = 0.028, *P* = 0.869; Figure 5I, *F*_treatment × session (2,30)_ = 0.829, *P* = 0.446, two-way RM ANOVA). The mice were re-exposed to the CPP apparatus 24 h later (test 2, without CNO treatment), and the CPP scores were similar among these groups (Figure 5D, test 2: *F*_treatment × compartment (1,30)_ = 0.106, *P* = 0.747; Figure 5H, test 2: *F*_treatment × compartment (1,15)_ = 0.011, *P* = 0.917, two-way RM ANOVA). These results showed that chemogenetic inhibition of AcbC-projecting vCA1 neurons, but not AcbC-projecting mPFC neurons, suppressed the preference for the cocaine-paired compartment in the memory retention test, indicating that AcbC-projecting vCA1 neurons, but not AcbC-projecting mPFC neurons, are essential for the retrieval of cocaine-CPP memory.

### The vCA1–AcbC Projections Are Required for the Retrieval of Cocaine-CPP Memory

**Figure 6:**
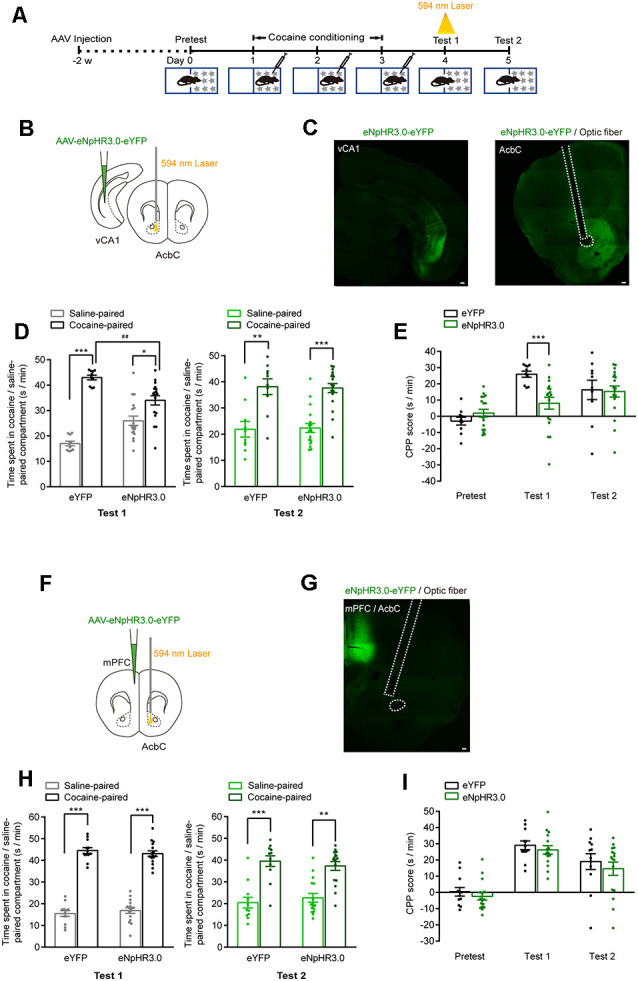
Optogenetic inhibition of vCA1–AcbC projection suppressed the retrieval of cocaine-CPP memory. **(A)** Behavioral schedule. *AAV-EF1α-eNpHR3.0-eYFP* was injected in the vCA1 or mPFC, and optical fiber was implanted in the AcbC 2 weeks before CPP training. A 594-nm yellow laser (5 mW, 5 min) was delivered during test 1. **(B,F)** Viral injection and optical fiber implantation. **(C,G)** Representative images of eNpHR3.0-eYFP fluorescence and location of optical fiber in the AcbC. Scale bar: 100 μm. **(D,H)** Bar graphs representing the time spent in the cocaine-paired and the saline-paired compartments of eYFP and eNpHR3.0 groups in the test 1 and test 2 sessions. *P < 0.05, **P < 0.01, ***P < 0.001 vs. saline-paired compartment, ^##^*P* < 0.01 vs. eYFP group. **(E,I)** Bar graphs showing the CPP scores of eYFP and eNpHR3.0 groups. ***P < 0.001 vs. eYFP group (vCA1–AcbC: eYFP *n* = 10, eNpHR3.0 *n* = 18; mPFC–AcbC: eYFP *n* = 12, eNpHR3.0 *n* = 16).

We then tested the role of vCA1–AcbC and mPFC–AcbC projections in the retrieval of cocaine-CPP memory (Figure 6A). *AAV-EF1α-eNpHR3.0-eYFP* was injected in the vCA1 or mPFC, and the optical fiber was implanted in the AcbC. The expression of eNpHR3.0-eYFP in the vCA1 or mPFC and the trace of optical fiber in the AcbC were detected (Figures 6B,C,F,G). The data showed that cocaine conditioning significantly increased the preference for the cocaine-paired compartment (Figures 6D,H). Optical stimulation during re-exposure to the CPP apparatus significantly decreased the duration in the cocaine-paired compartment in vCA1:eNpHR3.0 mice in memory retention test 1 (Figure 6D, test 1: *F*_treatment × compartment (1,26)_ = 12.315, *P* = 0.002; Figure 6E, *F*_treatment × session (2,52)_ = 6.147, *P* = 0.004, two-way RM ANOVA), while optical stimulation did not change the preference for the cocaine-paired compartment in mPFC:eNpHR3.0 mice (Figure 6H, test 1: *F*_treatment × compartment (1,26)_ = 0.557, *P* = 0.462; Figure 6I, *F*_treatment × session (2,52)_ = 0.04, *P* = 0.961, two-way RM ANOVA). The mice were re-exposed to the CPP apparatus 24 h later (test 2, without optical stimulation), and the CPP scores were similar among these groups (Figure 6D, test 2: *F*_treatment × compartment (1,26)_ = 0.025, *P* = 0.876; Figure 6H, test 2: *F*_treatment × compartment (1,26)_ = 0.489, *P* = 0.491, two-way RM ANOVA). The data above showed that the inhibition of vCA1–AcbC projection, but not mPFC–AcbC projection, suppressed memory retrieval, thus indicating that vCA1–AcbC projection is required for the retrieval of cocaine-CPP memory.

## Discussion

How is reward memory recalled, and what are the underlying neural circuits of memory retrieval? These are the critical questions of broad interests. In this study, we find that the vCA1 and AcbC act as hubs within a broader memory network for cocaine-CPP. We provide evidence that the vCA1 and its projection to the AcbC subserve the memory retrieval of cocaine-CPP. The mPFC–AcbC projection may not be involved in recent memory retrieval of cocaine-CPP.

Our previous work showed that vCA1 engram cells preferentially projected to the AcbC and encoded distinct contextual information and that the AcbC D1-expressing engram cells stored cocaine reward and associated context information. Cocaine-CPP evoked vCA1-engram-input-specific postsynaptic remodeling of the AcbC D1-engram that underlay the storage of cocaine-CPP memory (Zhou et al., 2019). The Acb receives dense projections from the vHipp, mPFC, BLA, and also paraventricular thalamus (PVT), but the neural circuits underlying drug memory retrieval remains unclear. In this study, we compared the roles of vCA1–AcbC and mPFC–AcbC projections in the recent memory retrieval of cocaine-CPP. We found that vCA1–AcbC projection, but not mPFC–AcbC projection, was critically involved in memory retrieval. The roles of other projections, such as BLA-AcbC and PVT-AcbC projections, in cocaine-CPP memory retrieval deserve further investigation.

Studies showed prominent labeling of afferents in the Acb shell (AcbSh) from the ventral hippocampus (vHipp), and most studies focused on the roles of glutamatergic innervation of the AcbSh in drug addiction. MacAskill et al. (2014) reported that cocaine repeated injections increased the D2/D1 ratio of vHipp inputs to the AcbSh. Britt et al. (2012) showed an increase in AMPAR/NMDAR ratio across vHipp-AcbSh synapses. The selective reversal of synaptic plasticity at mPFC-AchSh or vHipp-AcbSh synapses decreased response discrimination or response vigor during seeking, respectively (Pascoli et al., 2014). In this study, with anterograde tracing by the injection of self-complementary AAV (*scAAV*)*-hSyn-Cre* into the vCA1 of Ai14 mice, we found that vCA1 also extensively innervated the AcbC, so we compared the role of vCA1–AcbC and mPFC–AcbC projections in memory retrieval of cocaine-CPP by testing the effects of projection inhibition on the preference for the cocaine-paired side.

The core and the shell are the major nucleus accumbens subregions (AcbC and AcbSh) with respective afferent and efferent projections (Zahm and Brog, 1992) and distinct aspects of rewarding and motivational behavior (Mcfarland and Kalivas, 2001; Morgane et al., 2005). Biochemical studies showed that NMDA receptors within the AcbC, but not the AcbSh, were critically involved in restraint stress-induced reinstatement of cocaine-CPP (De Giovanni et al., 2016). The lesion studies suggested that the AcbC, but not AcbSh, played an essential role in learning and memory expression of cocaine self-administration, amphetamine-CPP, and food-CPP (Parkinson et al., 1999; Ito et al., 2004). However, the studies also showed a different result such that inhibition of PKMζ in the AcbC, not the AcbSh, abolished the memory retrieval of morphine-CPP (Li et al., 2011), but inhibition of PKMζ in the AcbSh, not the AcbC, impaired the memory retrieval of cocaine-CPP (Shabashov et al., 2012). It is hypothesized that the AcbC and AcbSh are implicated in distinct aspects of addictive behaviors (Kourrich et al., 2015), and the AcbSh is critical for the regulation of psychostimulant effects of cocaine and processing of irrelevant or non-rewarded information so that drugs may be obtained efficiently (Ito et al., 2004; Floresco, 2015). Given the dissociable contributions of the AcbC and AcbSh to addictive behaviors, the functions of the projections to the AcbC and AcbSh in drug addiction need further investigation.

It is known that the hippocampus, consistent with its critical roles in both cognitive and emotional domains, shows a marked variation along its dorsoventral axis (Bannerman et al., 2004; Mcnaughton and Corr, 2004; Fanselow and Dong, 2010; Li et al., 2018). It was hypothesized that the dorsal hippocampus (dHipp) was critical for spatial and contextual memory storage, while the vHipp mainly contributed to the regulation of anxiety-like behavior but not memory storage (Moser et al., 1995; Bannerman et al., 1999; Kheirbek et al., 2013). Inhibition of GABA_A_ receptor or GluR1 phosphorylation in the dHipp was required for memory formation of cocaine-CPP (Tropea et al., 2008; Hitchcock and Lattal, 2018). A lesion study showed that lesions limited to dHipp impaired, but lesions limited to vHipp enhanced, the learning and expression of cocaine-CPP (Ferbinteanu and Mcdonald, 2001). However, recent studies showed that the vHipp was also critical for memory storage. Ventral CA1 is a component of storage site for social memory (Okuyama et al., 2016) and contextual fear memory (Zhu et al., 2014). In the cocaine-CPP model, hippocampal place cells in the vCA1 drove the recruitment of D2-positive NAc medium spiny neurons and encoded the cocaine-paired location (Sjulson et al., 2018). The induction of LTP at vHipp–NAc synapse drove conditioned place preference and formed CPP memory (Legates et al., 2018). The present study showed that the inhibition of vCA1 neurons impaired the retrieval of cocaine-CPP memory, suggesting that vCA1 neurons are critically involved in the retrieval of reward associative memory, which may depend on the vCA1 neurotransmission onto the AcbC. The present study may help to support the necessity of mouse vCA1 and its projection to the AcbC for memory.

One recent work suggested that the chemogenetic inhibition of mPFC neurons impaired memory retrieval of a cocaine-CPP (Zhang et al., 2020). Zhang et al. (2020) used a training protocol with a non-alternative conditioning, while we used an alternative training protocol. They injected cocaine at a dose of 20 mg/kg, and we used 10 mg/kg. In their cocaine-CPP test, to examine the effects of chemogenetic inactivation of the mPFC neurons on cocaine-CPP expression, the mice received CNO (10 mg/kg, i.p.) 30 min before the test. In our study, the mice received CNO (1 mg/kg, i.p.) 30 min before the memory retention test. The discrepancy might attribute to the different doses of cocaine and CNO as well as different experimental conditions. The study by Otis et al. showed that cocaine conditioning-induced synaptic plasticity in the mPFC (prelimbic region) supported memory retrieval and that β-AR blockade reversed this plasticity and impaired memory retrieval (Otis and Mueller, 2017). This group also showed that the degree of intrinsic excitability of prelimbic neurons was correlated with the strength of memory retrieval (Otis et al., 2018). The study by Sun et al. (2019) showed that the membrane expression of GluA1 and GluA2 in the mPFC decreased following the recent memory retrieval, while the membrane expression of GluA1 and GluA2 in mPFC increased following the remote retrieval of morphine-associated memory. GluA2 endocytosis inhibition in the mPFC impaired the recent memory retrieval of morphine-CPP (Sun et al., 2019). These studies suggest complications of synaptic plasticity and innate excitability of mPFC neurons in the retrieval of drug-associative memory. Studies from Kalivas’ lab and others showed that the mPFC was critically involved in cue, stress, and drug priming-induced reinstatement of cocaine-SA (Capriles et al., 2003; Mcfarland et al., 2003; Di Pietro et al., 2006). In these studies, the drug memory should be updated and re-organized after 10-day extinction learning that was controlled by the mPFC. In addition, in the dorsal–ventral distinction within the mPFC in reinstatement behavior, it was found that the prelimbic cortex drove the expression of drug-seeking behaviors, whereas the infralimbic cortex suppressed reinstatement behaviors after extinction (Peters et al., 2008). Although no significant impairment of cocaine memory retrieval by chemogenetic inhibition of mPFC neurons was found in this study, some molecular changes were detected, such as increased pERK and c-Fos levels in the mPFC induced by memory retrieval. The effects of mPFC neuron inhibition on memory retrieval need further study by other means, and the dorsal–ventral distinction within the mPFC should be noted.

It was reported that 0.5 mg/kg CNO resulted in behavioral signs of seizure activity in approximately 20% of mice during fear conditioning (Garner et al., 2012). The high dose of CNO (10 mg/kg) also showed off-target effects (Martinez et al., 2019). A high dose of CNO may produce nonspecific side effects. Thus, we used CNO at a dose of 1 mg/kg CNO according to previous studies and found that CNO treatment 30 min before test 1 did not change cocaine-CPP memory retrieval in control mice, suggesting no detectable off-target effects of 1 mg/kg CNO in cocaine-CPP task. In our study, we found that chemogenetic inhibition of vCA1 and AcbC neurons greatly decreased the preference for the cocaine-paired side in the memory retention test, while the chemogenetic inhibition of mPFC neurons did not suppress cocaine-CPP memory retrieval. These results indicate that the inhibition of cocaine-CPP memory retrieval by the chemogenetic inhibition of vCA1 or AcbC neurons is not caused by the off-target effects of CNO.

Overall, our study demonstrates that the vCA1 and AcbC are recruited for the retrieval of cocaine-CPP memory and provides insights into vCA1–AcbC projection underlying cocaine reward memory retrieval.

## Data Availability Statement

The original contributions presented in the study are included in the article/Supplementary Materials, further inquiries can be directed to the corresponding author.

## Ethics Statement

The animal study was reviewed and approved by the National Institutes of Health Guide for the Care and Use of Laboratory Animals and Animal Care and Use Committee of Shanghai Medical College of Fudan University.

## Author Contributions

XL and LM designed the research. YZ and EY performed the research. HZ, ZL, XC, and DC performed the behavioral tests. XL and EY analyzed the data and wrote the article. All authors contributed to the article and approved the submitted version.

## Conflict of Interest

The authors declare that the research was conducted in the absence of any commercial or financial relationships that could be construed as a potential conflict of interest.

## Supplementary Material

SUPPLEMENTARY FIGURE 1 Anterograde labeling of Acb neurons innervated by vCA1. **(A)** Representative images of Acb neurons innervated by vCA1. The anterograde self-complementary AAV *(scAAV)-hSyn-Cre* was injected in the vCA1 of Ai14 mice. Scale bar: 100 μm. **(B)** Bar graph showing the percentage of AcbC and AcbSh neurons innervated by vCA1 in the Acb (*n* = 5).

## References

[B1] Bannerman D. M. Rawlins J. N. Mchugh S. B. Deacon R. M. Yee B. K. Bast T.. (2004). Regional dissociations within the hippocampus-memory and anxiety. Neurosci. Biobehav. Rev. 28, 273–283. 10.1016/j.neubiorev.2004.03.004 15225971

[B2] Bannerman D. M. Yee B. K. Good M. A. Heupel M. J. Iversen S. D. Rawlins J. N. (1999). Double dissociation of function within the hippocampus: a comparison of dorsal, ventral and complete hippocampal cytotoxic lesions. Behav. Neurosci. 113, 1170–1188. 10.1037/0735-7044.113.6.1170 10636297

[B3] Barr J. L. Shi X. Zaykaner M. Unterwald E. M. (2020). Glycogen synthase kinase 3β in the ventral hippocampus is important for cocaine reward and object location memory. Neuroscience 425, 101–111. 10.1016/j.neuroscience.2019.10.055 31783102 PMC7297259

[B4] Berhow M. T. Hiroi N. Nestler E. J. (1996). Regulation of ERK (extracellular signal regulated kinase), part of the neurotrophin signal transduction cascade, in the rat mesolimbic dopamine system by chronic exposure to morphine or cocaine. J. Neurosci. 16, 4707–4715. 10.1523/JNEUROSCI.16-15-04707.1996 8764658 PMC6579030

[B5] Berke J. D. Hyman S. E. (2000). Addiction, dopamine and the molecular mechanisms of memory. Neuron 25, 515–532. 10.1016/s0896-6273(00)81056-9 10774721

[B6] Boening J. A. (2001). Neurobiology of an addiction memory. J. Neural Transm. 108, 755–765. 10.1007/s007020170050 11478425

[B7] Britt J. P. Benaliouad F. Mcdevitt R. A. Stuber G. D. Wise R. A. Bonci A. (2012). Synaptic and behavioral profile of multiple glutamatergic inputs to the nucleus accumbens. Neuron 76, 790–803. 10.1016/j.neuron.2012.09.040 23177963 PMC3607383

[B8] Capriles N. Rodaros D. Sorge R. E. Stewart J. (2003). A role for the prefrontal cortex in stress- and cocaine-induced reinstatement of cocaine seeking in rats. Psychopharmacology 168, 66–74. 10.1007/s00213-002-1283-z 12442201

[B9] Chen R. H. Sarnecki C. Blenis J. (1992). Nuclear localization and regulation of erk- and rsk-encoded protein kinases. Mol. Cell. Biol. 12, 915–927. 10.1128/mcb.12.3.915 1545823 PMC369523

[B10] Cohen S. Greenberg M. E. (2008). Communication between the synapse and the nucleus in neuronal development, plasticity and disease. Annu. Rev. Cell Dev. Biol. 24, 183–209. 10.1146/annurev.cellbio.24.110707.175235 18616423 PMC2709812

[B11] Corbit L. H. Muir J. L. Balleine B. W. (2001). The role of the nucleus accumbens in instrumental conditioning: evidence of a functional dissociation between accumbens core and shell. J. Neurosci. 21, 3251–3260. 10.1523/JNEUROSCI.21-09-03251.2001 11312310 PMC6762583

[B12] Crombag H. S. Jedynak J. P. Redmond K. Robinson T. E. Hope B. T. (2002). Locomotor sensitization to cocaine is associated with increased Fos expression in the accumbens, but not in the caudate. Behav. Brain Res. 136, 455–462. 10.1016/s0166-4328(02)00196-1 12429408

[B13] Cruz F. C. Javier Rubio F. Hope B. T. (2015). Using c-fos to study neuronal ensembles in corticostriatal circuitry of addiction. Brain Res. 1628, 157–173. 10.1016/j.brainres.2014.11.005 25446457 PMC4427550

[B14] De Giovanni L. N. Guzman A. S. Virgolini M. B. Cancela L. M. (2016). NMDA antagonist MK 801 in nucleus accumbens core but not shell disrupts the restraint stress-induced reinstatement of extinguished cocaine-conditioned place preference in rats. Behav. Brain Res. 315, 150–159. 10.1016/j.bbr.2016.08.011 27506656

[B15] Di Pietro N. C. Black Y. D. Kantak K. M. (2006). Context-dependent prefrontal cortex regulation of cocaine self-administration and reinstatement behaviors in rats. Eur. J. Neurosci. 24, 3285–3298. 10.1111/j.1460-9568.2006.05193.x 17156389

[B16] Everitt B. J. Wolf M. E. (2002). Psychomotor stimulant addiction: a neural systems perspective. J. Neurosci. 22, 3312–3320. 10.1523/JNEUROSCI.22-09-03312.2002 11978805 PMC6758398

[B17] Fanselow M. S. Dong H. W. (2010). Are the dorsal and ventral hippocampus functionally distinct structures? Neuron 65, 7–19. 10.1016/j.neuron.2009.11.031 20152109 PMC2822727

[B18] Ferbinteanu J. Mcdonald R. J. (2001). Dorsal/ventral hippocampus, fornix and conditioned place preference. Hippocampus 11, 187–200. 10.1002/hipo.1036 11345125

[B19] Flagel S. B. Clark J. J. Robinson T. E. Mayo L. Czuj A. Willuhn I.. (2011). A selective role for dopamine in stimulus-reward learning. Nature 469, 53–57. 10.1038/nature09588 21150898 PMC3058375

[B20] Floresco S. B. (2015). The nucleus accumbens: an interface between cognition, emotion and action. Annu. Rev. Psychol. 66, 25–52. 10.1146/annurev-psych-010213-115159 25251489

[B21] Garner A. R. Rowland D. C. Hwang S. Y. Baumgaertel K. Roth B. L. Kentros C.. (2012). Generation of a synthetic memory trace. Science 335, 1513–1516. 10.1126/science.1214985 22442487 PMC3956300

[B22] Goto Y. Grace A. A. (2008). Limbic and cortical information processing in the nucleus accumbens. Trends Neurosci. 31, 552–558. 10.1016/j.tins.2008.08.002 18786735 PMC2884964

[B23] Hitchcock L. N. Lattal K. M. (2018). Involvement of the dorsal hippocampus in expression and extinction of cocaine-induced conditioned place preference. Hippocampus 28, 226–238. 10.1002/hipo.22826 29341327 PMC5916867

[B24] Hyman S. E. (2005). Addiction: a disease of learning and memory. Am. J. Psychiatry 162, 1414–1422. 10.1176/appi.ajp.162.8.1414 16055762

[B25] Ito R. Robbins T. W. Everitt B. J. (2004). Differential control over cocaine-seeking behavior by nucleus accumbens core and shell. Nat. Neurosci. 7, 389–397. 10.1038/nn1217 15034590

[B26] Kelley A. E. (2004). Memory and addiction: shared neural circuitry and molecular mechanisms. Neuron 44, 161–179. 10.1016/j.neuron.2004.09.016 15450168

[B27] Kheirbek M. A. Drew L. J. Burghardt N. S. Costantini D. O. Tannenholz L. Ahmari S. E.. (2013). Differential control of learning and anxiety along the dorsoventral axis of the dentate gyrus. Neuron 77, 955–968. 10.1016/j.neuron.2012.12.038 23473324 PMC3595120

[B28] Kourrich S. Calu D. J. Bonci A. (2015). Intrinsic plasticity: an emerging player in addiction. Nat. Rev. Neurosci. 16, 173–184. 10.1038/nrn3877 25697160

[B29] Legates T. A. Kvarta M. D. Tooley J. R. Francis T. C. Lobo M. K. Creed M. C.. (2018). Reward behaviour is regulated by the strength of hippocampus-nucleus accumbens synapses. Nature 564, 258–262. 10.1038/s41586-018-0740-8 30478293 PMC6292781

[B30] Li K. Shen S. Ji Y. T. Li X. Y. Zhang L. S. Wang X. D. (2018). Melatonin augments the effects of fluoxetine on depression-like behavior and hippocampal BDNF-TrkB signaling. Neurosci. Bull. 34, 303–311. 10.1007/s12264-017-0189-z 29086908 PMC5856716

[B31] Li Y. Q. Xue Y. X. He Y. Y. Li F. Q. Xue L. F. Xu C. M.. (2011). Inhibition of PKMζ in nucleus accumbens core abolishes long-term drug reward memory. J. Neurosci. 31, 5436–5446. 10.1523/JNEUROSCI.5884-10.2011 21471379 PMC3150199

[B32] MacAskill A. F. Cassel J. M. Carter A. G. (2014). Cocaine exposure reorganizes cell type- and input-specific connectivity in the nucleus accumbens. Nat. Neurosci. 17, 1198–1207. 10.1038/nn.3783 25108911 PMC4146520

[B33] Martinez V. K. Saldana-Morales F. Sun J. J. Zhu P. J. Costa-Mattioli M. Ray R. S. (2019). Off-target effects of clozapine-*N*-oxide on the Chemosensory reflex are masked by high stress levels. Front. Physiol. 10:521. 10.3389/fphys.2019.00521 31178741 PMC6538678

[B34] Mcfarland K. Kalivas P. W. (2001). The circuitry mediating cocaine-induced reinstatement of drug-seeking behavior. J. Neurosci. 21, 8655–8663. 10.1523/JNEUROSCI.21-21-08655.2001 11606653 PMC6762812

[B35] Mcfarland K. Lapish C. C. Kalivas P. W. (2003). Prefrontal glutamate release into the core of the nucleus accumbens mediates cocaine-induced reinstatement of drug-seeking behavior. J. Neurosci. 23, 3531–3537. 10.1523/JNEUROSCI.23-08-03531.2003 12716962 PMC6742291

[B36] Mcnaughton N. Corr P. J. (2004). A two-dimensional neuropsychology of defense: fear/anxiety and defensive distance. Neurosci. Biobehav. Rev. 28, 285–305. 10.1016/j.neubiorev.2004.03.005 15225972

[B37] Mizoguchi H. Yamada K. Mizuno M. Mizuno T. Nitta A. Noda Y.. (2004). Regulations of methamphetamine reward by extracellular signal-regulated kinase 1/2/ets-like gene-1 signaling pathway *via* the activation of dopamine receptors. Mol. Pharmacol. 65, 1293–1301. 10.1124/mol.65.5.1293 15102958

[B38] Morgane P. J. Galler J. R. Mokler D. J. (2005). A review of systems and networks of the limbic forebrain/limbic midbrain. Prog. Neurobiol. 75, 143–160. 10.1016/j.pneurobio.2005.01.001 15784304

[B39] Moser M. B. Moser E. I. Forrest E. Andersen P. Morris R. G. (1995). Spatial learning with a minislab in the dorsal hippocampus. Proc. Natl. Acad. Sci. U S A 92, 9697–9701. 10.1073/pnas.92.21.9697 7568200 PMC40869

[B40] Okuyama T. Kitamura T. Roy D. S. Itohara S. Tonegawa S. (2016). Ventral CA1 neurons store social memory. Science 353, 1536–1541. 10.1126/science.aaf7003 27708103 PMC5493325

[B41] Otis J. M. Dashew K. B. Mueller D. (2013). Neurobiological dissociation of retrieval and reconsolidation of cocaine-associated memory. J. Neurosci. 33, 1271a–1281a. 10.1523/JNEUROSCI.3463-12.2013 23325262 PMC3564635

[B42] Otis J. M. Fitzgerald M. K. Yousuf H. Burkard J. L. Drake M. Mueller D. (2018). Prefrontal neuronal excitability maintains cocaine-associated memory during retrieval. Front. Behav. Neurosci. 12:119. 10.3389/fnbeh.2018.00119 29962941 PMC6010542

[B43] Otis J. M. Mueller D. (2017). Reversal of cocaine-associated synaptic plasticity in medial prefrontal cortex parallels elimination of memory retrieval. Neuropsychopharmacology 42, 2000–2010. 10.1038/npp.2017.90 28466871 PMC5561348

[B44] Parkinson J. A. Olmstead M. C. Burns L. H. Robbins T. W. Everitt B. J. (1999). Dissociation in effects of lesions of the nucleus accumbens core and shell on appetitive pavlovian approach behavior and the potentiation of conditioned reinforcement and locomotor activity by D-amphetamine. J. Neurosci. 19, 2401–2411. 10.1523/JNEUROSCI.19-06-02401.1999 10066290 PMC6782569

[B45] Pascoli V. Terrier J. Espallergues J. Valjent E. O’Connor E. C. Luscher C. (2014). Contrasting forms of cocaine-evoked plasticity control components of relapse. Nature 509, 459–464. 10.1038/nature13257 24848058

[B46] Pennartz C. M. Ito R. Verschure P. F. Battaglia F. P. Robbins T. W. (2011). The hippocampal-striatal axis in learning, prediction and goal-directed behavior. Trends Neurosci. 34, 548–559. 10.1016/j.tins.2011.08.001 21889806

[B47] Peters J. Lalumiere R. T. Kalivas P. W. (2008). Infralimbic prefrontal cortex is responsible for inhibiting cocaine seeking in extinguished rats. J. Neurosci. 28, 6046–6053. 10.1523/JNEUROSCI.1045-08.2008 18524910 PMC2585361

[B48] Phillips P. E. Stuber G. D. Heien M. L. Wightman R. M. Carelli R. M. (2003). Subsecond dopamine release promotes cocaine seeking. Nature 422, 614–618. 10.1038/nature01476 12687000

[B49] Phillipson O. T. Griffiths A. C. (1985). The topographic order of inputs to nucleus accumbens in the rat. Neuroscience 16, 275–296. 10.1016/0306-4522(85)90002-8 4080159

[B50] Pierce R. C. Pierce-Bancroft A. F. Prasad B. M. (1999). Neurotrophin-3 contributes to the initiation of behavioral sensitization to cocaine by activating the Ras/Mitogen-activated protein kinase signal transduction cascade. J. Neurosci. 19, 8685–8695. 10.1523/JNEUROSCI.19-19-08685.1999 10493769 PMC6783001

[B51] Russo S. J. Nestler E. J. (2013). The brain reward circuitry in mood disorders. Nat. Rev. Neurosci. 14, 609–625. 10.1038/nrn3381 23942470 PMC3867253

[B52] Sesack S. R. Grace A. A. (2010). Cortico-basal ganglia reward network: microcircuitry. Neuropsychopharmacology 35, 27–47. 10.1038/npp.2009.93 19675534 PMC2879005

[B53] Shabashov D. Shohami E. Yaka R. (2012). Inactivation of PKMζ in the NAc shell abolished cocaine-conditioned reward. J. Mol. Neurosci. 47, 546–553. 10.1007/s12031-011-9671-7 22127928

[B54] Sjulson L. Peyrache A. Cumpelik A. Cassataro D. Buzsaki G. (2018). Cocaine place conditioning strengthens location-specific hippocampal coupling to the nucleus accumbens. Neuron 98, 926.e5–934.e5. 10.1016/j.neuron.2018.04.015 29754750 PMC6154491

[B55] Spencer S. Brown R. M. Quintero G. C. Kupchik Y. M. Thomas C. A. Reissner K. J.. (2014). α2δ-1 signaling in nucleus accumbens is necessary for cocaine-induced relapse. J. Neurosci. 34, 8605–8611. 10.1523/JNEUROSCI.1204-13.2014 24948814 PMC4061396

[B56] Stuber G. D. Klanker M. De Ridder B. Bowers M. S. Joosten R. N. Feenstra M. G.. (2008). Reward-predictive cues enhance excitatory synaptic strength onto midbrain dopamine neurons. Science 321, 1690–1692. 10.1126/science.1160873 18802002 PMC2613864

[B57] Sun X. Wang N. Wang X. Sun L. Li Y. Cui C. (2019). AMPA receptor in ventromedial prefrontal cortex plays different roles in the recent and remote retrieval of morphine-associated memory. Neurochem. Res. 44, 1939–1949. 10.1007/s11064-019-02827-z 31209728

[B58] Tropea T. F. Kosofsky B. E. Rajadhyaksha A. M. (2008). Enhanced CREB and DARPP-32 phosphorylation in the nucleus accumbens and CREB, ERK and GluR1 phosphorylation in the dorsal hippocampus is associated with cocaine-conditioned place preference behavior. J. Neurochem. 106, 1780–1790. 10.1111/j.1471-4159.2008.05518.x 18554320 PMC2706979

[B59] Valjent E. Corvol J. C. Pages C. Besson M. J. Maldonado R. Caboche J. (2000). Involvement of the extracellular signal-regulated kinase cascade for cocaine-rewarding properties. J. Neurosci. 20, 8701–8709. 10.1523/JNEUROSCI.20-23-08701.2000 11102476 PMC6773075

[B60] Wang Y. Zhang H. Cui J. Zhang J. Yin F. Guo H.. (2019). Opiate-associated contextual memory formation and retrieval are differentially modulated by dopamine D1 and D2 signaling in hippocampal-prefrontal connectivity. Neuropsychopharmacology 44, 334–343. 10.1038/s41386-018-0068-y 29728647 PMC6300561

[B61] Xu C. M. Wang J. Wu P. Xue Y. X. Zhu W. L. Li Q. Q.. (2011). Glycogen synthase kinase 3β in the nucleus accumbens core is critical for methamphetamine-induced behavioral sensitization. J. Neurochem. 118, 126–139. 10.1111/j.1471-4159.2011.07281.x 21517846

[B62] Xu C. M. Wang J. Wu P. Zhu W. L. Li Q. Q. Xue Y. X.. (2009). Glycogen synthase kinase 3β in the nucleus accumbens core mediates cocaine-induced behavioral sensitization. J. Neurochem. 111, 1357–1368. 10.1111/j.1471-4159.2009.06414.x 19799712

[B63] Zahm D. S. Brog J. S. (1992). On the significance of subterritories in the “accumbens” part of the rat ventral striatum. Neuroscience 50, 751–767. 10.1016/0306-4522(92)90202-d 1448200

[B64] Zhang T. Yanagida J. Kamii H. Wada S. Domoto M. Sasase H.. (2020). Glutamatergic neurons in the medial prefrontal cortex mediate the formation and retrieval of cocaine-associated memories in mice. Addict. Biol. 25:e12723. 10.1111/adb.12723 30734456

[B65] Zhou Y. Zhu H. Liu Z. Chen X. Su X. Ma C.. (2019). A ventral CA1 to nucleus accumbens core engram circuit mediates conditioned place preference for cocaine. Nat. Neurosci. 22, 1986–1999. 10.1038/s41593-019-0524-y 31719672

[B66] Zhu H. Pleil K. E. Urban D. J. Moy S. S. Kash T. L. Roth B. L. (2014). Chemogenetic inactivation of ventral hippocampal glutamatergic neurons disrupts consolidation of contextual fear memory. Neuropsychopharmacology 39, 1880–1892. 10.1038/npp.2014.35 24525710 PMC4059896

