# Supplementary figures and images for: The Projection From Ventral CA1, Not Prefrontal Cortex, to Nucleus Accumbens Core Mediates Recent Memory Retrieval of Cocaine-Conditioned Place Preference
Source: Front Behav Neurosci. 2020 Nov 16;14:558074. doi: 10.3389/fnbeh.2020.558074 (PMC7701212; doi:10.3389/fnbeh.2020.558074)

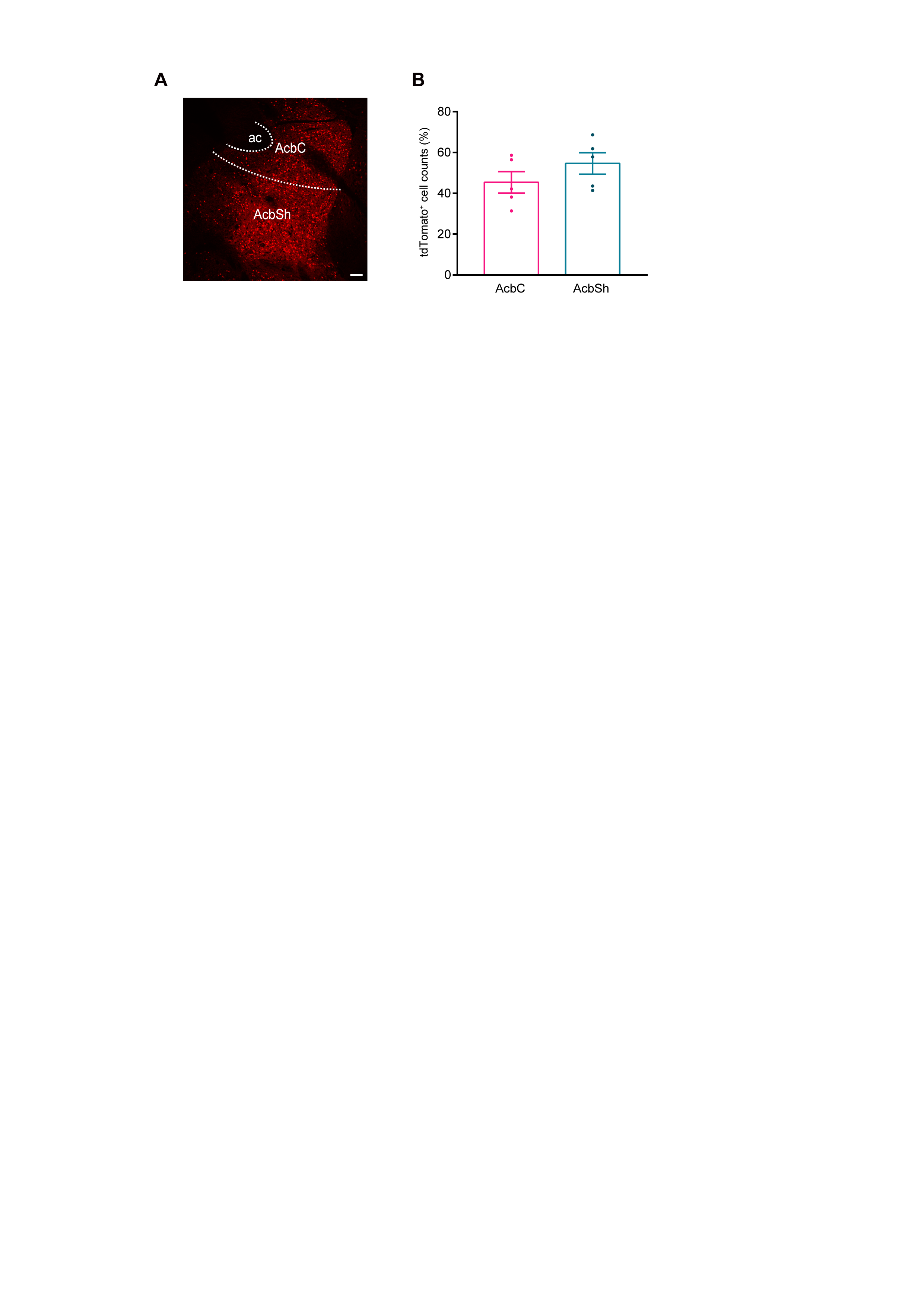

Supplement: SUPPLEMENTARY FIGURE 1 — Anterograde labeling of Acb neurons innervated by vCA1. (A) Representative images of Acb neurons innervated by vCA1. The anterograde self-complementary AAV (scAAV)-hSyn-Cre was injected in the vCA1 of Ai14 mice. Scale bar: 100 μm. (B) Bar graph showing the percentage of AcbC and AcbSh neurons innervated by vCA1 in the Acb (n = 5). [file Image_1.TIF]
